# New diagnosis of conversion disorder following anterior lumbar interbody fusion: a case report

**DOI:** 10.1093/jscr/rjad341

**Published:** 2023-06-16

**Authors:** Brendan P Mitchell, Jake M Bianco, Faith M G Kim, M Camden Whitaker

**Affiliations:** Department of Orthopaedic Surgery, University of Kansas School of Medicine - Wichita, Wichita, KS, USA; Department of Orthopaedic Surgery, University of Kansas School of Medicine - Wichita, Wichita, KS, USA; University of Kansas School of Medicine - Wichita, Wichita, KS, USA; Department of Orthopaedic Surgery, University of Kansas School of Medicine - Wichita, Wichita, KS, USA

**Keywords:** anterior lumbar interbody fusion, conversion disorder, lumbar fusion, post-operative neurologic deficit

## Abstract

Neurologic deficit after lumbar spine surgery is a rare and serious complication that must be promptly diagnosed and treated to avoid long-term neurologic disability. Anterior lumbar interbody fusion (ALIF) is an effective technique for the treatment of recurrent disc herniation and lumbar disc degeneration. This case report describes a 20-year-old female with L5-S1 recurrent disc herniation and lumbar degeneration. She underwent an L5-S1 ALIF complicated by post-operative lower left extremity paralysis. Revision surgery with downsizing of the ALIF cage was performed with normal neuromonitoring throughout the procedure. The patient displayed persistent post-operative neurologic deficits despite no evidence of central or foraminal compression. Patient was later diagnosed with conversion disorder by neurology during her hospitalization. This case report presents the initial diagnosis of conversion disorder after a routine ALIF procedure, which led to surgical re-exploration and prolonged inpatient hospital stay. Psychiatric diagnoses must be considered when neurologic deficits are present with no apparent organic cause.

## INTRODUCTION

Anterior lumbar interbody fusion (ALIF) is an increasingly popular technique for the treatment of lumbosacral pathologies, with a 168% increase from 2007 to 2014 [1, 2]. Indications for ALIF include spondylolisthesis, degenerative disc disease, recurrent disc herniation, pseudoarthrosis and adjacent segment disease [3, 4].

The most common complications associated with ALIF include vascular injury, neurologic injury, post-operative ileus, surgical site infection and retrograde ejaculation [5]. The rate of nerve root injuries associated with ALIF is low, and it is theorized to occur as a result of stretch neuropraxia secondary to a significant increase in disc height [6].

In this manuscript, we present a case report of a 20-year-old female who developed conversion paralysis after a single-level anterior lumber interbody fusion.

## CASE REPORT

A 20-year-old female presented to our clinic with a chief complaint of low back, buttock and lower left extremity pain. She had previously undergone an L5 hemilaminectomy and L5 microdiscectomy 16 and 13 months prior, respectively, with a surgeon unaffiliated with our institution, without improvement. She also recently failed 11 months of conservative treatment. The patient had no history of medical or psychiatric problems.

**Figure 1:**
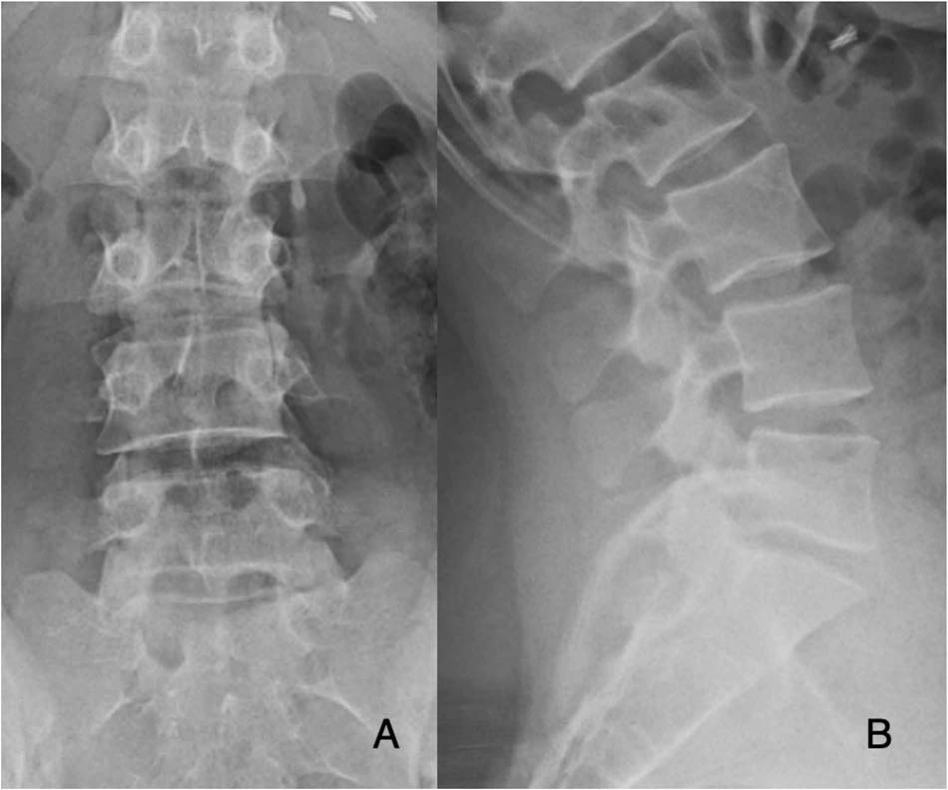
Preoperative PA and lateral films demonstrate decreased disc height at L5-S1.

**Figure 2:**
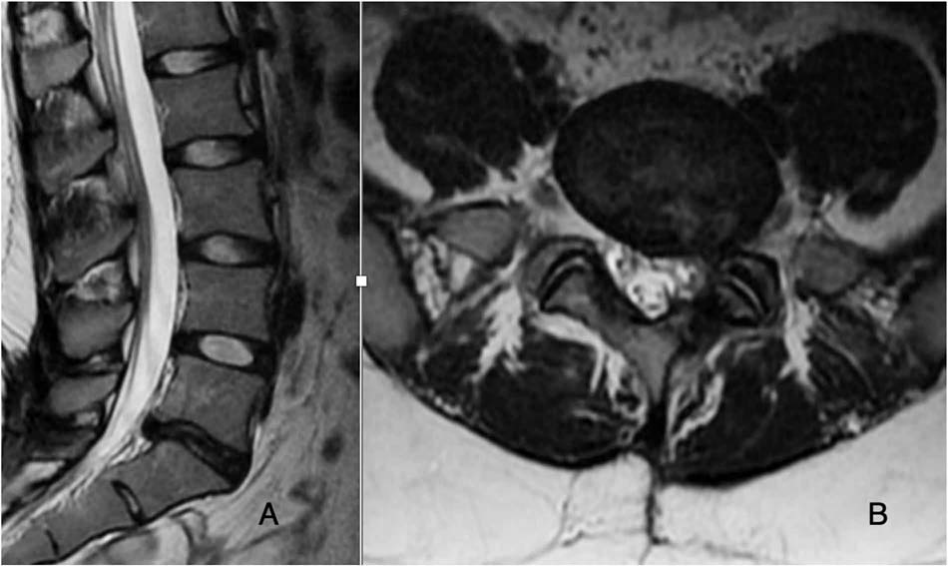
T2-weighted MRI sagittal and axial cuts demonstrate recurrent disc herniation with left-sided foraminal stenosis and degenerative changes at L5-S1.

Preoperatively, her physical exam demonstrated left-sided quadriceps weakness and minimally diminished sensation in an L3-L5 distribution. The patient’s magnetic resonance imaging (MRI) demonstrated degeneration of the L5-S1 disc with disc height loss and left-sided L5-S1 foraminal stenosis (Figs 1 and 2).

She was taken to the operating room and underwent an L5-S1 ALIF through a left-sided paramedian approach. A size 12 Medtronic Divergence PEEK lordotic cage was used (Medtronic, Dublin IE) and no intra-operative complications or excessive blood loss was noted.

Approximately 90 minutes after the procedure, the patient began to experience increased numbness and weakness in the lower left extremity. Examination of the left lower extremity revealed 2/5 strength to ankle plantarflexion and dorsiflexion, big toe extension and 5/5 strength to knee flexion and extension. She exhibited intact sensation on the medial and lateral aspect of her thigh as well as on the dorsum and lateral aspect of her foot; however, she had nearly absent sensation to the medial, first dorsal webspace, and plantar aspects of the left foot. Sacral hypesthesia and urinary or stool incontinence were absent.

Emergent MRI was obtained, which demonstrated successful indirect decompression of the exiting L5 nerve root with no evidence of new compression of the thecal sac or malposition of the interbody cage.

Fearing excessive traction on the L5-S1 nerve roots, the patient was brought back to the operating room emergently for exploration and revision ALIF with downsizing of the ALIF cage. Transcranial motor-evoked potentials and electromyography was normal pre-incision through closing.

On post-operative day 1, sensation had improved to near normal in all dermatomes, and strength had improved to 4/5 in all muscle groups. She ambulated normally and was discharged.

She returned to the emergency department 2 days later with complaints of worsening lower left extremity numbness and weakness. A thorough neurologic examination revealed absent motor function in the L2-S2 distribution with a positive Hoover sign as well as numbness in a ‘stocking glove’ distribution. Neurology consultants diagnosed conversion disorder after a thorough medical workup, and follow-up with outpatient psychiatry was arranged.

**Figure 3:**
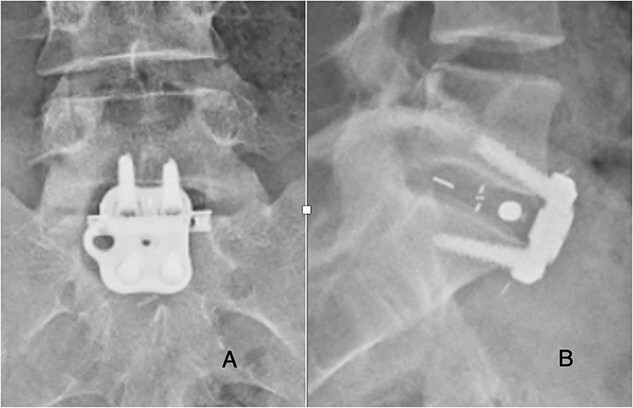
Post-operative posterior-anterior (**A**) and lateral (**B**) films demonstrate ALIF cage and plate placement.

She was followed until 12 weeks post-operatively, with complete resolution of symptoms and did not appear for any appointments past 12 weeks. Radiographs obtained at follow-up demonstrated stable cage and plate placement (Fig. 3).

## DISCUSSION

Post-operative neurologic deficits are a rare complication following ALIF [7]. Although rare, these complications can be devastating and should be recognized promptly. Conversion disorder, also known as functional neurologic disorder, is defined as the presence of neurologic symptoms that are inconsistent with structural neurologic findings consistent with other established neurologic clinical diagnoses. Symptoms can present as nonepileptic seizures, visual disturbances, movement disorders and even paralysis. Although the aetiology is unknown, pathogenesis involves many patient factors that may predispose patients to conversion disorder. Risk factors include female gender, younger age, prior history of psychiatric disorders and any type of emotional or physical trauma. Conversion disorder is usually a diagnosis of exclusion and should only be diagnosed after a thorough medical and surgical evaluation have excluded any other diagnoses that may mimic a patient’s symptoms [8].

Psychiatric disorders have risen in patients undergoing spinal fusions and laminectomies. These procedures have been shown to be risk factors for perioperative adverse events and post-operative complications [9]. Symptoms of paralysis in the absence of normal neuromonitoring and positive Hoover sign in our patient are indicative of a possible conversion paralysis, and this was confirmed by the neurology team at our institution after ruling out organic causes.

## Data Availability

The data that support the findings of this study are available on request from the corresponding author, BPM. The data are not publicly available due to restrictions e.g. their containing information that could compromise the privacy of research participants.
